# Impact of bedside lung ultrasound on physician clinical decision-making in an emergency department in Nepal

**DOI:** 10.1186/s12245-020-00273-1

**Published:** 2020-04-03

**Authors:** Darlene R. House, Yogendra Amatya, Benjamin Nti, Frances M. Russell

**Affiliations:** 1 Department of General Practice and Emergency Medicine, Patan Academy of Health Sciences, Kathmandu, Nepal; 2 Department of Emergency Medicine, Indiana University School of Medicine, 720 Eskenazi Avenue, Indianapolis, IN 46202 USA

**Keywords:** Lung ultrasound, Clinical decision-making, Nepal, Diagnosis, Developing countries

## Abstract

**Background:**

Lung ultrasound is an effective tool for the evaluation of undifferentiated dyspnea in the emergency department. Impact of lung ultrasound on clinical decisions for the evaluation of patients with dyspnea in resource-limited settings is not well-known. The objective of this study was to evaluate the impact of lung ultrasound on clinical decision-making for patients presenting with dyspnea to an emergency department in the resource-limited setting of Nepal.

**Methods:**

A prospective, cross-sectional study of clinicians working in the Patan Hospital Emergency Department was performed. Clinicians performed lung ultrasounds on patients presenting with dyspnea and submitted ultrasounds with their pre-test diagnosis, lung ultrasound interpretation, post-test diagnosis, and any change in management.

**Results:**

Twenty-two clinicians participated in the study, completing 280 lung ultrasounds. Diagnosis changed in 124 (44.3%) of patients with dyspnea. Clinicians reported a change in management based on the lung ultrasound in 150 cases (53.6%). Of the changes in management, the majority involved treatment (83.3%) followed by disposition (13.3%) and new consults (2.7%).

**Conclusions:**

In an emergency department in Nepal, bedside lung ultrasound had a significant impact on physician clinical decision-making, especially on patient diagnosis and treatment.

## Background

Chronic obstructive pulmonary disease (COPD) and lower respiratory tract infection are two of the top five leading causes of death and disability in Nepal, with respiratory complaints being a leading cause of presentation to the emergency department [1]. With a high burden of respiratory diseases and difficulty obtaining radiographs, lung ultrasound has the potential to be an effective tool for use in a resource-limited setting [2]. In patients presenting to the emergency department with undifferentiated dyspnea, lung ultrasound has been shown to be an effective tool for expediting diagnosis and changing acute management [3–8]. Utilization of lung ultrasound for clinical care of patients with respiratory distress has been shown to save time, reduce cost, and improve clinical diagnosis and management in several high and middle-income countries [4, 6, 8–11]. In resource-limited settings, lung ultrasound is particularly useful as alternative imaging modalities may be unavailable, difficult to obtain, or too expensive [12].

Few studies have been performed in resource-limited settings to evaluate the impact of ultrasound on clinical decisions, and these studies have focused primarily on the general use of point-of-care ultrasound [12–15]. While Reynolds et al. evaluated bedside ultrasound overall, they also reported data for each individual study type, finding that lung ultrasound changed management in 45% of cases in Tanzania [12]. To our knowledge, no studies have specifically evaluated the impact of lung ultrasound on management of dyspneic patients in a low-income country.

The objective of this study was to evaluate the impact of lung ultrasound on clinical decision-making of physicians caring for patients presenting with dyspnea in an emergency department in Nepal.

## Methods

### Study design

A prospective cross-sectional study was performed from August 2017 through May 2019 at Patan Hospital Emergency Department (ED). We received ethical approval from the Nepal Health Research Council Ethical Review Board. Written informed consent was obtained from each participant.

### Study setting and population

Patan Hospital is a government academic institution with a 35-bed ED and an annual volume of approximately 48,000 patients. Medical officers, physicians who have completed medical school and preparing for post-graduate medical education, provide the majority of care in the ED. They are supervised by faculty who have either completed general practice or emergency medicine (EM) training.

Lung ultrasounds were performed and interpreted by physicians, including medical officers, EM fellows and faculty, working in the Patan Hospital ED. Physicians had 8 h of lung ultrasound training that consisted of a 1-hour lecture, followed by hands-on practice on a human model and then 4 h of one-on-one, hands-on lung ultrasound scans in the ED with a credentialed ultrasonographer to ensure a standardized method of acquiring and interpreting images using the bedside lung ultrasound in emergency (BLUE) protocol [4]. When physicians demonstrated ability to consistently acquire images and apply the BLUE protocol, they began to scan independently and submit scans for the study. Demographics of the physicians participating in the study were collected, including position and years of experience.

### Study protocol

While providing care for patients with acute dyspnea during clinical shifts, physicians completed lung ultrasounds using a SonoSite M Turbo (Fujifilm SonoSite, Inc.) and the curvilinear probe. Ultrasounds were performed on a convenience sample of patients, both adults and children. Sonographers used a previously described 10-zone scanning protocol, assessing for lung sliding, A lines, B lines, consolidations, or pleural effusions [16, 17]. A lines were defined as recurrent horizontal echogenic artifacts arising from the pleural line generated by sub-pleural air [4]. B lines were defined as discrete vertical hyperechoic artifacts arising from the pleural line, extending all the way to the bottom of the ultrasound screen, erasing A lines, and moving with lung sliding [4]. Consolidation was defined as the presence of > 2 focal B-lines or a sub-pleural hypoechoic or tissue-like area with B-lines at the far-field border [4].

A standardized data collection form was used to report their pre-test diagnosis, ultrasound findings, followed by post-test diagnosis, utilizing the bedside lung ultrasound in emergency (BLUE) protocol for interpretation [4]. Pre-test diagnosis was based on history and physical exam findings, and recorded prior to lung ultrasound being performed. Post-test diagnosis incorporated lung ultrasound findings and interpretation. Participants recorded how ultrasound may have impacted acute management according to four categories: disposition (i.e., change in level of care, admission versus discharge), treatment (ordered new medication, changed fluids, performed or did not perform a procedure such as chest tube or thoracentesis), new consultation, or other. Each ultrasound was recorded and reviewed by one of two expert sonographers with registered diagnostic medical sonographer certification and > 1000 previously performed ultrasounds. Experts were blinded to the bedside clinician’s interpretation and patient information. Ten percent of images were randomly selected for blind review by a second expert reviewer to assess inter-rater reliability between the experts.

### Statistical analysis

Descriptive statistics were used to identify percentage of ultrasounds that affected diagnosis and management. Kappa analysis was performed to evaluate inter-rater reliability between the clinician’s and expert’s ultrasound interpretations.

## Results

Twenty-two clinicians participated in the study, submitting 280 lung ultrasounds (Table 1). Inter-rater reliability for ultrasound interpretations between the experts was 0.9 and between the clinician and expert was 0.8, representing excellent agreement.

**Table 1:** Demographics

Provider	No. of participants (*N* = 22)	No. of ultrasounds performed (*N* = 280)
Faculty	2 (9%)	17 (6%)
EM fellow	2 (9%)	51 (18%)
Medical officer	18 (82%)	212 (76%)

Pre-test diagnosis remained the same after ultrasound in 156 (55.7%) patients. Pre-test diagnosis changed based on ultrasound findings in 124 cases (44.3%), see Table 2.

**Table 2:** Pre- and post-test diagnoses

Pre-test diagnosis	Post-test diagnosis	*N*
Change in diagnosis		124 (44.3%)
Pneumonia	No pneumonia: viral or other febrile illness	58
Pneumonia	COPD	10
COPD	Pneumonia	28
COPD	Heart failure	4
Enteric fever	Pneumonia	2
Asthma	Pneumonia	3
Heart failure	COPD	2
Heart failure	Pneumonia	5
Viral respiratory illness	Pneumonia	3
Pleural effusion	Pneumonia	4
Pleural effusion	Normal	1
Pneumothorax	Normal	2
Pulmonary contusion	Normal	2
No change in diagnosis		156 (55.7%)
Pneumonia	Pneumonia	110
COPD	COPD	14
Asthma	Asthma	2
Heart failure	Heart failure	12
Viral respiratory illness	Viral respiratory illness	12
Pneumothorax	Pneumothorax	3
End stage renal disease/fluid overload	End stage renal disease/fluid overload	2
Pleural effusion	Pleural effusion	1

Clinicians reported a change in management based on ultrasound in 150 cases (53.6%). The changes included treatment management in 125 of 150 cases (83.3%), disposition in 20 cases (13.3%), and obtaining a new consult in 4 cases (2.7%). One documented “other” as their change in management, stating the need for further investigation for cause of patient’s dyspnea.

Of the 125 cases with reported changes in treatment, clinicians documented the specific change in treatment in 104 cases: change in antibiotic administration (antibiotics canceled in 56 cases and antibiotics given in 41 cases), furosemide administration (two cases), and change in procedure (thoracentesis performed in five cases, thoracentesis not needed in two cases, and two cases did not need chest tube placement).

## Discussion

This study demonstrates the significant impact bedside lung ultrasound had on clinical decision-making in an urban government academic ED in Nepal. The majority of impact was on diagnostic impression, with 45% of patients having a change in diagnosis, and subsequent treatment of patients, with over 50% having a change in acute management after ultrasound was performed.

Bedside ultrasound had a similar impact on clinical decision-making in Tanzania, where Reynolds et al. found that of all the ultrasounds evaluated in their study, lung ultrasound had one of the highest impacts on decision-making in their emergency department, impacting decisions in 45% of patients evaluated [12]. In our study, lung ultrasound leads to a change in management in 53% of patients. This slightly higher percentage may be attributed to the difference in the training level of providers performing the ultrasounds between the two locations. The majority of ultrasounds performed in our study were completed by medical officers who may be more likely to rely on ultrasound findings for help with clinical decisions than emergency medicine residents or physicians with more clinical experience.

This data suggests that lung ultrasound is an effective bedside tool that can be utilized by providers to help guide patient care, especially in resource-limited settings. Frequently, patients have to pay prior to investigations and be stabilized before they can go to radiology for chest imaging. In Nepal, obtaining a chest x-ray for patients with dyspnea can take 2 h on average [2]. These long delays in obtaining chest imaging then lead to delays in disease-targeted interventions. Further training and implementation of bedside lung ultrasound for patients with acute dyspnea may decrease delays in management and improve patient care.

Our study has several limitations. Similar to Reynolds et al., we relied on clinician self-reporting regarding changes in diagnosis and management [12]. Self-reporting could be biased towards clinicians reporting those ultrasounds in which they felt more confident and could lead to an over-estimation of impact. Despite this, the clinicians’ had substantial changes in their pre- and post-test diagnoses, which had significant agreement with the expert’s blinded interpretations. Additionally, data was missing regarding some of the clinician’s specific change in management; however, the data reported and changes in diagnosis provide a reflection of the various ways ultrasound impacts management in this setting. As this was a study of clinician decision-making, we did not collect patient demographic data and follow patients through their emergency department and hospital stay to further evaluate impact on patient care. Further studies should aim to follow patients throughout their ED and hospital stay with structured follow-up to objectively measure outcomes related to changes in management based on ultrasound findings.

## Conclusions

In the low-income setting of Nepal, bedside lung ultrasound had a significant impact on physician clinical decision-making, especially on patient diagnosis and treatment.

## Data Availability

The datasets used and/or analyzed during the current study are available from the corresponding author on reasonable request.

## Abbreviations

BLUE: Bedside lung ultrasound in emergency
ED: Emergency department
COPD: Chronic obstructive pulmonary disease

## References

[1] Institute for Health Metrics and Evaluation: Nepal 2019 [Available from: http://www.healthdata.org/nepal.

[2] Amatya Y, Rupp J, Russell FM, Saunders J, Bales B, House DR (2018). Diagnostic use of lung ultrasound compared to chest radiograph for suspected pneumonia in a resource-limited setting. Int J Emerg Med..

[3] Gallard E, Redonnet JP, Bourcier JE, Deshaies D, Largeteau N, Amalric JM (2015). Diagnostic performance of cardiopulmonary ultrasound performed by the emergency physician in the management of acute dyspnea. Am J Emerg Med..

[4] Lichtenstein DA (2014). Lung ultrasound in the critically ill. Ann Intensive Care..

[5] Mantuani D, Nagdev A (2013). Three-view bedside ultrasound to differentiate acute decompensated heart failure from chronic obstructive pulmonary disease. Am J Emerg Med.

[6] Pirozzi C, Numis FG, Pagano A, Melillo P, Copetti R, Schiraldi F (2014). Immediate versus delayed integrated point-of-care-ultrasonography to manage acute dyspnea in the emergency department. Crit Ultrasound J..

[7] Russell FM, Ehrman RR, Cosby K, Ansari A, Tseeng S, Christain E (2015). Diagnosing acute heart failure in patients with undifferentiated dyspnea: a lung and cardiac ultrasound (LuCUS) protocol. Acad Emerg Med..

[8] Guttikonda SNR, Vadapalli K (2018). Approach to undifferentiated dyspnea in emergency department: aids in rapid clinical decision-making. Int J Emerg Med..

[9] Jones BP, Tay ET, Elikashvili I, Sanders JE, Paul AZ, Nelson BP (2016). Feasibility and safety of substituting lung ultrasonography for chest radiography when diagnosing pneumonia in children: a randomized controlled trial. Chest..

[10] Wang XT, Liu DW, Zhang HM, Chai WZ (2014). Integrated cardiopulmonary sonography: a useful tool for assessment of acute pulmonary edema in the intensive care unit. J Ultrasound Med..

[11] Xirouchaki N, Kondili E, Prinianakis G, Malliotakis P, Georgopoulos D (2014). Impact of lung ultrasound on clinical decision making in critically ill patients. Intensive Care Med..

[12] Reynolds TA, Amato S, Kulola I, Chen CJ, Mfinanga J, Sawe HR (2018). Impact of point-of-care ultrasound on clinical decision-making at an urban emergency department in Tanzania. PLoS One..

[13] Kotlyar S, Moore CL (2008). Assessing the utility of ultrasound in Liberia. J Emerg Trauma Shock..

[14] Shah SP, Epino H, Bukhman G, Umulisa I, Dushimiyimana JM, Reichman A (2009). Impact of the introduction of ultrasound services in a limited resource setting: rural Rwanda 2008. BMC Int Health Hum Rights..

[15] Henwood PC, Mackenzie DC, Liteplo AS, Rempell JS, Murray AF, Leo MM (2017). Point-of-care ultrasound use, accuracy, and impact on clinical decision making in Rwanda hospitals. J Ultrasound Med..

[16] Cortellaro F, Colombo S, Coen D, Duca PG (2012). Lung ultrasound is an accurate diagnostic tool for the diagnosis of pneumonia in the emergency department. Emerg Med J..

[17] Lichtenstein DA, Lascols N, Meziere G, Gepner A (2004). Ultrasound diagnosis of alveolar consolidation in the critically ill. Intensive Care Med..

